# “Loop-10” line-assisted clip closure method: closure of perforation in re-do peroral endoscopic myotomy

**DOI:** 10.1016/j.vgie.2023.01.007

**Published:** 2023-03-09

**Authors:** Haruhiro Inoue, Yuto Shimamura, Mai Fukuda, Ryohei Ando, Hisaki Kato, Mayo Tanabe, Marc Julius Navarro

**Affiliations:** 1 Digestive Diseases Center, Showa University Koto Toyosu Hospital, Tokyo, Japan; 2 Digestive Diseases Center, Showa University Koto Toyosu Hospital, Tokyo, Japan; 3 Institute of Digestive and Liver Diseases, St. Luke’s Medical Center, Quezon City, Philippines

## Abstract

Video 1 Demonstration of the Loop-10 closure technique for unintentional mucosal perforation during re-do esophageal peroral endoscopic myotomy.

Demonstration of the Loop-10 closure technique for unintentional mucosal perforation during re-do esophageal peroral endoscopic myotomy.

## Background

Complete closure of mucosal defect is essential, especially when a full-layer resection is intentionally performed or when we encounter unintentional tissue perforation. A standard method for closing a large mucosal defect has not yet been established. It is technically demanding to close a large-size tissue defect solely by endoscopic clips. A line-assisted clip closure technique has been reported to overcome technical difficulties.^1^, ^2^, ^3^, ^4^, ^5^, ^6^, ^7^, ^8^ In principle, by pulling a thread anchored to the first clip, the succeeding clips can be efficiently and effectively applied to approximate mucosal edges with adequate tissue traction. However, the remaining issue seen in the line-assisted clip closure method is the detachment of the line upon completion of the defect closure. Some authors have reported the use of the following accessories to detach to the line: endoscope surgical scissors, loop cutter, and argon plasma coagulation probe.^1^, ^2^, ^3^, ^4^, ^5^, ^6^, ^7^, ^8^ These accessories are not widely and readily available in some centers and will add to procedure expense. To solve the remaining issue regarding detachment of the thread in line-assisted closure, we developed the “Loop-10” closure technique.

## Case

A 37-year-old female patient presented to us with a diagnosed case of achalasia, nonsigmoid, type I, who underwent esophageal peroral endoscopic myotomy (POEM) in our institution 3 years previously. At the interval follow-ups after the POEM procedure, the patient claimed to have improved and be in remission with a reported Eckardt score of 0. Three years after the POEM procedure, the patient claimed gradual occasional recurrence of dysphagia, retrosternal pain, and regurgitation, with a reported Eckardt score of 3 to 4; repeat timed-barium esophagogram revealed recurrence of some narrowing at the level of gastroesophageal junction. The patient was diagnosed to have recurrent achalasia and was primed for possible treatment options, such as balloon dilation and re-do POEM. The patient opted to undergo a re-do POEM procedure.

**Figure 1:**
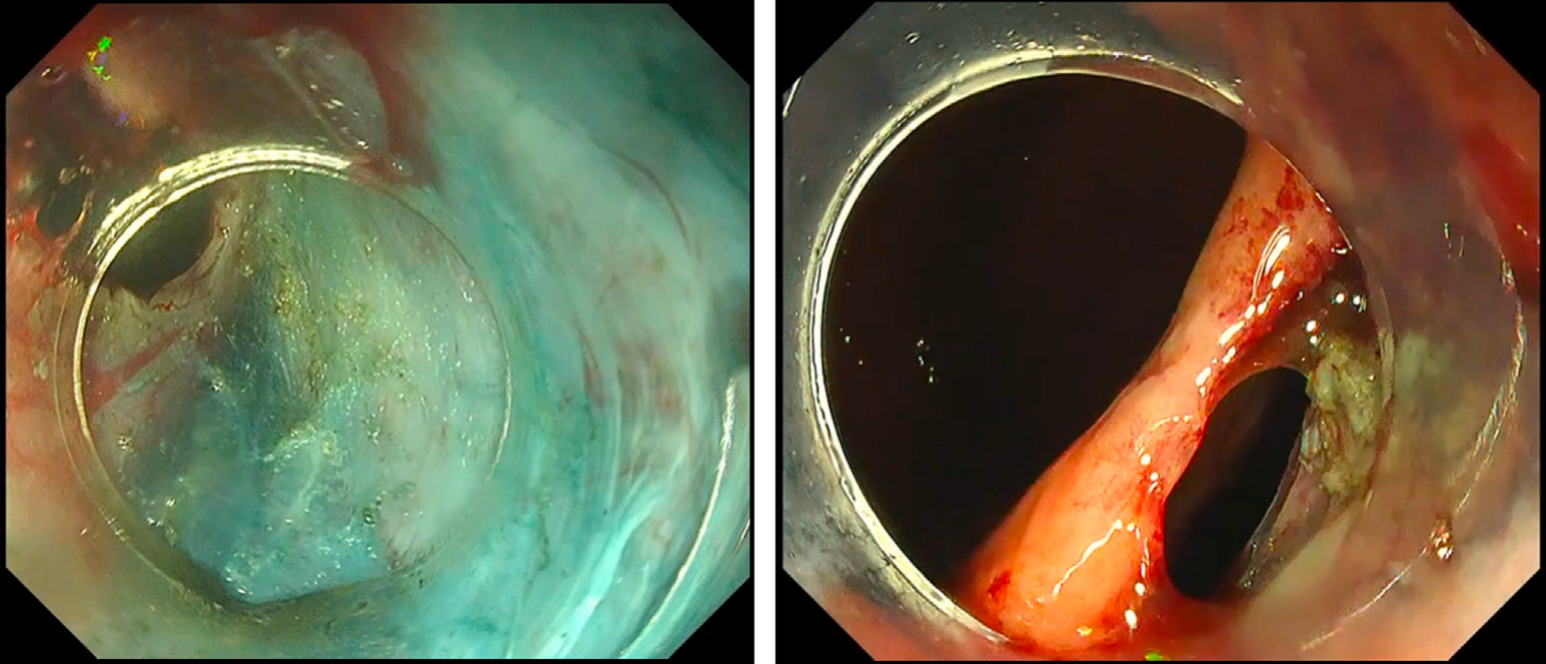
Unintentional mucosal perforation at the level of gastroesophageal junction: submucosal tunnel view and luminal view.

During the re-do POEM, while doing submucosal tunneling, severe fibrosis was encountered at the level of the gastroesophageal junction. Careful dissection of the limited submucosal space expansion was done. However, in an attempt to bypass the area of fibrosis, unintentional mucosal perforation was encountered (Fig. 1). After the completion of submucosal tunneling and selective myotomy, to avoid postoperative adverse events, we opted to close the unintentional mucosal perforation by the Loop-10 technique (Video 1, available online at www.giejournal.org).

## Procedure

### Construction of the First Clip With Loop-10

**Figure 2:**
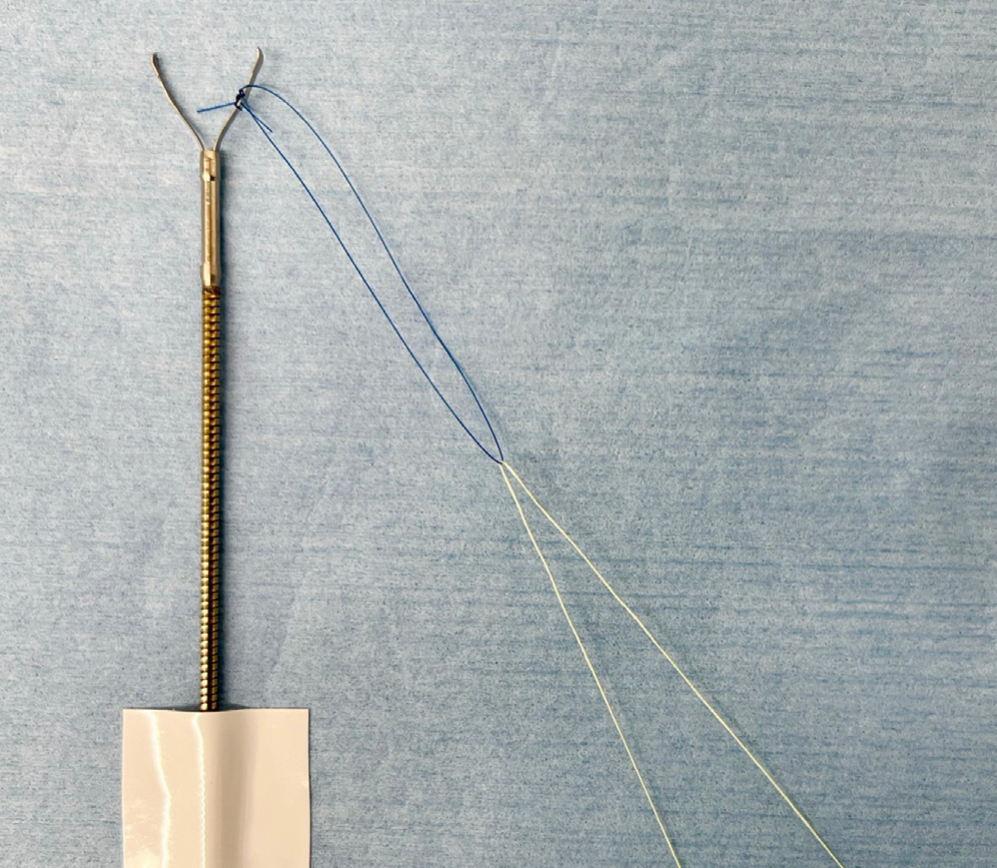
Anatomy of first endoscopic clip with Loop-10.

We created the main loop using surgical suture (5-0 Nylon Suture, GA05NA; NescoSuture, Qingdao City, China), according to the approximate longitudinal length of the defect. Then, we attached the created main loop to 1 arm of the repositionable endoscopic clip. For this case demonstration, we fixed the main loop using an endoscopic clip without an arm window (QuickClip Pro, HX-202LR; Olympus, Center Valley, Pa, USA), but using an endoscopic clip with an arm window (SureClip Plus, ROCC-F-26; Micro-Tech Endoscopy, Ann Arbor, Mich, USA) was not a problem. Afterward, we hooked the support thread using monofilament nylon line to the main loop, with length approximately longer than the length of our therapeutic endoscope’s (H290T; Olympus) accessory channel (Fig. 2).

### Proper Loop-10 Closure Technique

**Figure 3:**
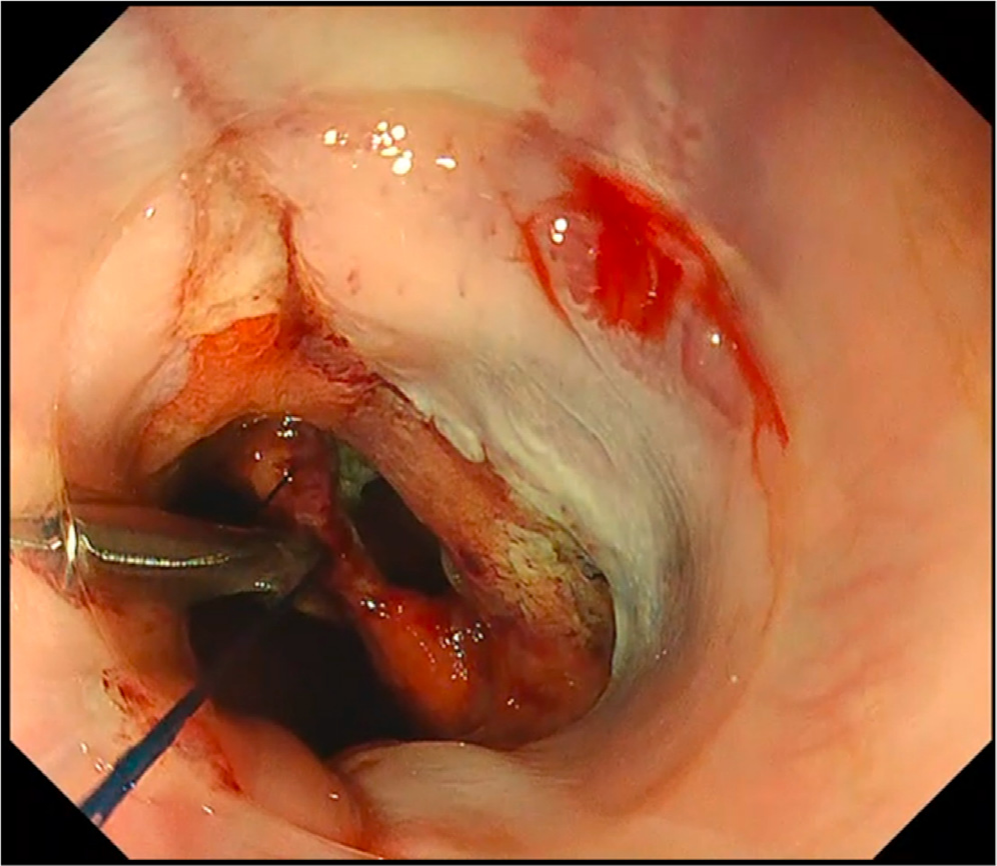
Proper Loop-10 closure technique: deployment of first endoscopic clip with Loop-10.

**Figure 4:**
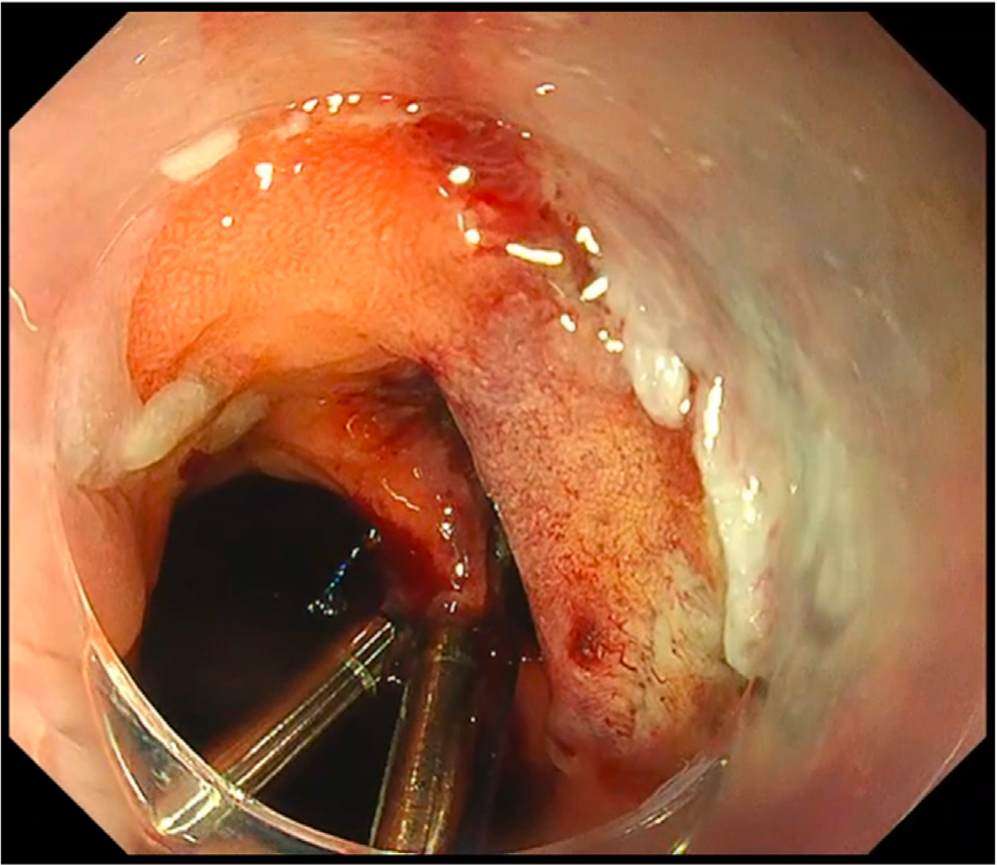
Proper Loop-10 closure technique: deployment of the succeeding endoscopic clips.

**Figure 5:**
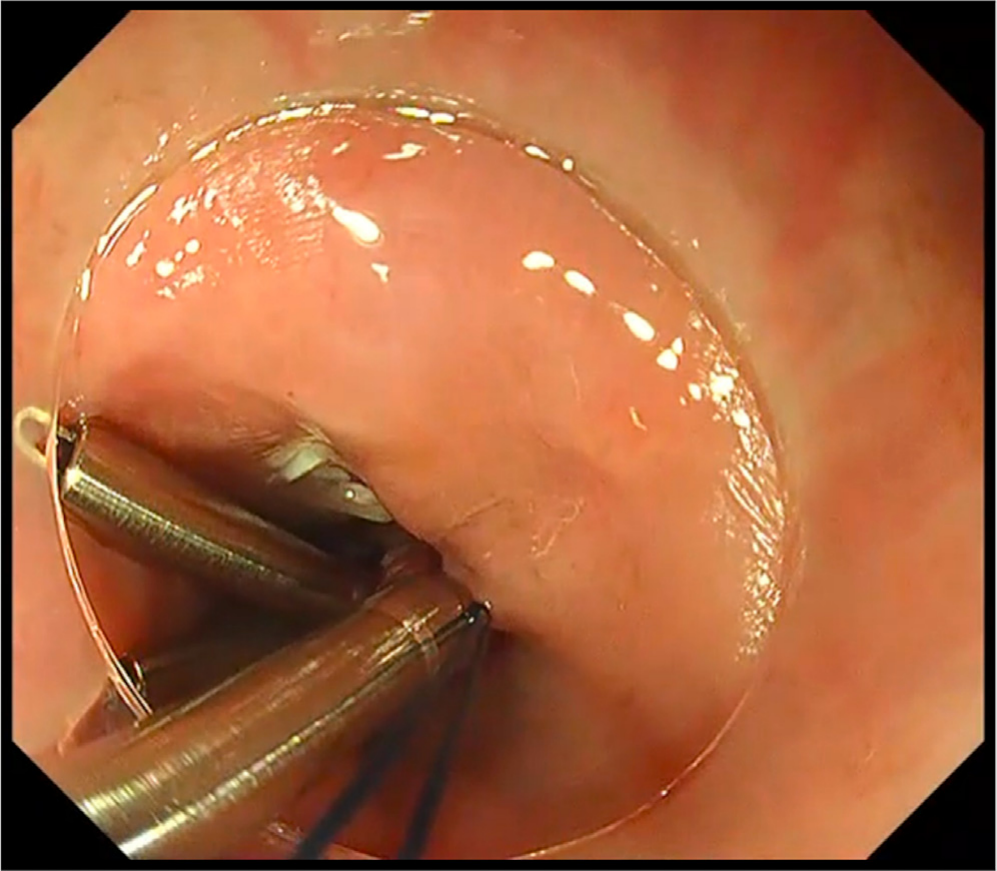
Proper Loop-10 closure technique: complete closure of the defect.

**Figure 6:**
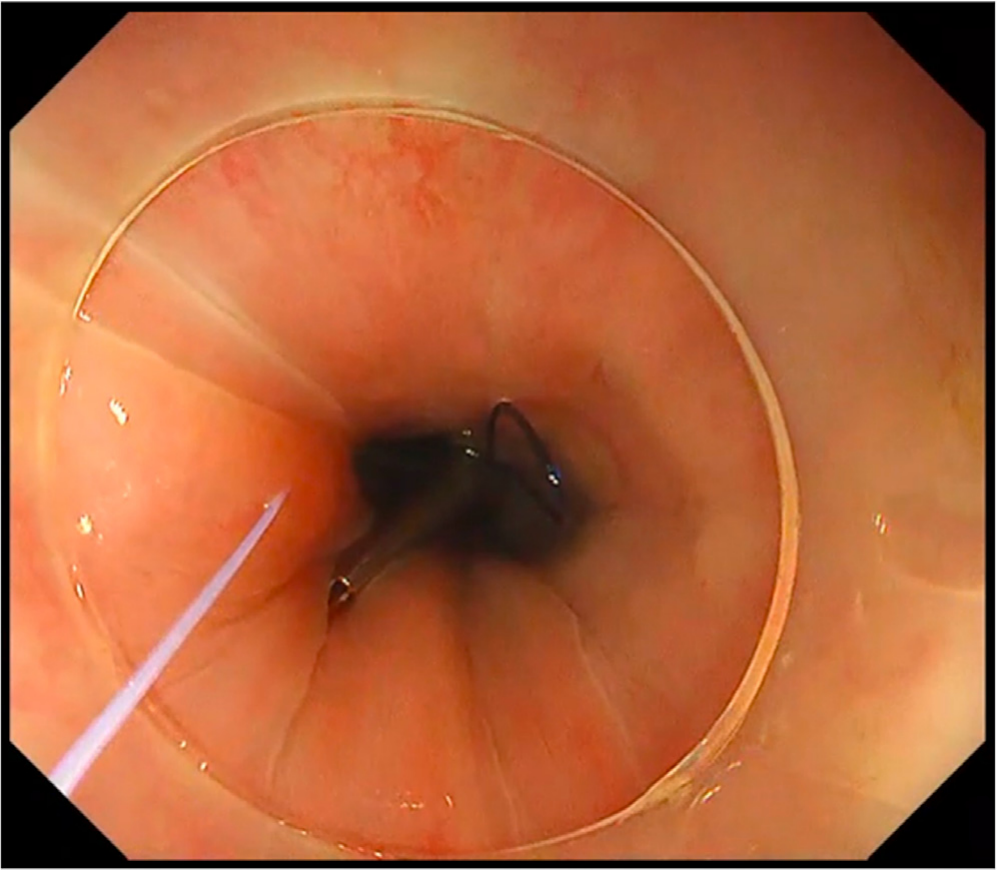
Proper Loop-10 closure technique: removal of the support thread.

We deployed the constructed first clip with Loop-10 at the most distal part of the defect (Fig. 3). The assistant then provided adequate traction and lift by pulling the support thread anchored to the main loop. Then, the succeeding endoscopic clips were deployed to ride on the main loop (Fig. 4), until full closure of the defect was achieved (Fig. 5). For the succeeding endoscopic clips, any preferred and appropriate through-the-scope clips (single-use or repositionable, with or without arm window) are suitable for this closure technique. For this case demonstration, we used repositionable endoscopic clips without arm window (QuickClip Pro, HX-202LR, Olympus, Tokyo, Japan). Afterward, removal of the support thread was done by carefully pulling one end of the support thread (Fig. 6).

## Outcome

Second-look endoscopy and repeat timed-barium esophagogram 1 day after the patient’s procedure confirmed complete and sustained closure of the unintentional mucosal perforation using our Loop-10 closure technique.

## Disclosure

*Dr Inoue is an advisor for Olympus Corporation and Top Corporation. All other authors disclosed no financial relationships.*

## Supplementary data

Video 1 Demonstration of the Loop-10 closure technique for unintentional mucosal perforation during re-do esophageal peroral endoscopic myotomy.
